# Therapeutic Notes

**Published:** 1896-06-27

**Authors:** 


### THERAPEUTIC NOTES.

### ANTISEPTICS FOR INFANT DIARRHŒA.

The treatment of infant diarrhœa so often resolves itself into the routine practice of giving small doses of grey powder, either with or without the addition of some form of astringent, that a short summary of some of the later methods may be of value. Soltau Fenwick, in communication to the *British Medical Journal*[1] on the subject of infant diarrhœa, remarks that "during the last three years I have used various medicinal antiseptics in more than 500 cases of digestive disorders in children. . . . The results which have accrued from their use in cases of infantile diarrhœa have been so satisfactory that for more than twelve months I have never had to resort either to astringents or to opium." In chronic diarrhœa due to fermentation he recommends for infants three to four weeks old three grams of resorcin every four hours; large doses appear to be well tolerated, and the drug readily taken by infants. Should the condition be of long standing, with the probable complication of follicular ulceration of the large intestine, antiseptics of the nature of resorcin or calomel, which are chiefly operative on the acid contents of the stomach and duodenum, should be supplemented by antiseptics which are active in the alkaline portions of the intestine, namely, benzol-naphthol, salol, and salicylate of bismuth. In a recent paper by Dauchez[2] the following plan is recommended when the diarrhœa is dependent on follicular ulceration of the large intestine: Large injections of a weak antiseptic solution should be passed into the intestine by means of a long catheter—from one and a-half to two pints once or twice a day. The injection may be made of a solution of hyposulphite of sodium, or of tincture of benzoin and water.

Dauchez contends that the success of this treatment largely depends on the removal of decomposing and irritative food *débris*, and for this purpose care must be taken that the injection reaches far up the large intestine. To obtain this end the right thigh should be raised so as to render the cæcum as dependent as possible, and thus facilitate the flow of the solution by gravity.

In similar cases tannigen[3] has been vaunted almost as a specific. It is a diacetyl derivative of tannin, which undergoes no decomposition in the stomach, and consequently does not interfere either directly or indirectly with gastric digestion. Its value, probably, depends entirely on the astringent action of the nascent tannin in the intestine.

It must, however, be remembered that tannigen is practically useless when the diarrhœa depends chiefly on fermentative action in the stomach. The latter must first be treated by appropriate dieting, and by gastric antiseptics such as resorcin.

To sum up: In the early stages, characterised by gastric fermentation, repeated small doses of calomel (¼ to 1-6th gr.) or resorcin (gr. 4) should be tried. In later stages, in which intestinal fermentation is coexistent, benzol napthol (3 gr.), salol (gr. 5), salicylate of bismuth (gr. 5), should be given in addition. In the final stage, with follicular ulceration of the large intestine, resort must be had to Dauchez's method by injection, or to small doses of tannigen.

[1] B.M.J., Dec. 2, 1895. [2] Rev. des mal de l'enfant, May, 1896. [3] Therap. Monatsch., Sept, 1894. B.M.J. Epit., Sept, 1894. Wein Clinic Kind., 1895.

